# Unexpectedly broad target recognition of the CRISPR-mediated virus defence system in the archaeon *Sulfolobus solfataricus*

**DOI:** 10.1093/nar/gkt767

**Published:** 2013-09-09

**Authors:** Andrea Manica, Ziga Zebec, Julia Steinkellner, Christa Schleper

**Affiliations:** Department of Genetics in Ecology, University of Vienna, Althanstrasse 14, A-1090 Vienna, Austria

## Abstract

The hyperthermophilic archaeon *Sulfolobus solfataricus* carries an extensive array of clustered regularly interspaced short palindromic repeats (CRISPR) systems able to mediate DNA degradation of invading genetic elements when complementarity to the small CRISPR-derived (cr)RNAs is given. Studying virus defence *in vivo* with recombinant viral variants, we demonstrate here that an unexpectedly high number of mutations are tolerated between the CRISPR-derived guide RNAs (crRNAs) and their target sequences (protospacer). Up to 15 mismatches in the crRNA still led to ∼50% of DNA degradation, when these mutations were outside the ‘seed’ region. More than 15 mutations were necessary to fully abolished interference. Different from other CRISPR systems investigated *in vivo*, mutations outside the protospacer region indicated no need for a protospacer adjacent motif sequence to confer DNA interference. However, complementarity of only 3 nucleotides between the repeat-derived 5′ handle of the crRNA and nucleotides adjacent to the protospacer enabled self-recognition, i.e. protection of the host locus. Our findings show commonalities and differences among the various CRISPR-mediated defence systems and suggest that they should not merely be perceived as a ‘first-barrier-defence system’ but may be considered to have a broader mechanism that allows host cells to cope with viruses keeping them at reduced levels.

## INTRODUCTION

Recently, a new defence mechanism that protects bacteria and archaea from invading genetic elements through an RNA-mediated DNA interference mechanism was discovered. On challenge of a virus or plasmid, the host cells integrate small DNA sequences of 30–45 nt of the invader genome into their chromosome (the spacers), in a region called clustered regularly interspaced short palindromic repeats (CRISPR). After transcription of the CRISPR region, the full-length RNA is processed into small CRISPR RNAs (crRNAs) that mediate degradation of a complementary invading DNA with the help of protein complexes encoded adjacent to the CRISPR locus, called CRISPR-associated proteins (Cas) (1–4). This defence mechanism is present in most archaea (87%) and in >45% of bacteria (CRISPRdb http://crispr.u-psud.fr/crispr/) and shows a great diversity (5). CRISPR loci can be divided into >45 different families based on the presence of particular Cas protein sets, which are now classified into three major types: I, II and III (6–8).

The CRISPR locus is transcribed in unique orientation, from a promoter region within the leader sequence (2), although the presence of antisense transcripts has also been detected (9–11). Processing of the full-length CRISPR transcript has been studied in several archaeal and bacterial systems (9,12,13). It is performed by a Cas protein belonging to the Cas6 family in the CRISPR types I and III, or by a mechanism involving non-coding antisense RNA (tracrRNAs) and cellular RNAseIII in type II CRISPR systems (14). In the type I and III CRISPR-Cas systems studied so far, the crRNA contains an 8-nt tag at its 5′ terminus (5′ handle) corresponding to 8 nucleotides of the repeat preceding the spacer, followed by the unique spacer sequence, then followed by a 3′ sequence corresponding to the remaining part of the next direct repeat (15,16). The 5′ tag was shown to be responsible for self-/non–self-discrimination of the target sequence in the bacterium *Staphylococcus epidermidis*, protecting the genomic CRISPR locus from degradation by the self-originated crRNA (17). The authors identified the positions important in the protospacer adjacent sequence (PAS) that can block CRISPR activity when matching the central 4 nt of the 5′ handle of the spacer, demonstrating how the CRISPR system can protect its own locus by the targeting of self-originated spacers.

New insights into the nature of the interaction between the crRNA and its DNA target sequence were recently gained by Wiedenheft *et al.* in *Pseudomonas aeruginosa* and by Semenova *et al.* in *Escherichia coli*, who provided evidence that in bacterial type I systems the first 8 nt of the 5′ region of the spacer are critical for target recognition (acting like a ‘seed’ sequence) and that mutations in the corresponding region of the protospacer have a major impact on the DNA interference activity (18,19), while four mutations between the spacer and target outside the seed region were tolerated (18). This observation differs from the conclusion drawn by Deveau *et al.* in *Streptococcus thermophilus* where even a single mutation in the protospacer sequence abolished completely the CRISPR defence mechanism (20). The protospacer adjacent motif (PAM) sequences were first detected by Mojica *et al.* as short dinucleotide sequences that have an important role in new spacer acquisition (21). These short motifs are often located between the first nucleotides at the 3′-end of the protospacer and determine in which orientation the protospacer will be inserted into the CRISPR locus. Successive studies have also demonstrated the influence of this region during the interference process in some systems, such that even a single mutation in the PAM sequence can abolish DNA interference (3,18,20).

We and others have recently established an *in vivo* study system for the hyperthermophilic archaeon *S. solfataricus* (3,22). This organism possesses a rather complex and extended CRISPR-Cas assembly with several Cas modules classified into the CRISPR-Cas system type I, type IIIA and IIIB (8). The activity of three of the six *S. solfataricus* CRISPR loci (9,23) were previously investigated *in vivo* (3,22). While CRISPR E-F were inactive due to the lack of transcription and the absence of correctly processed crRNAs (3), loci A-B and C-D produced active crRNAs and these were able to reduce transfection efficiency of a virion (22) or plasmid (3) carrying protospacers with high similarity to the respective crRNA. From those studies it appeared that in *S. solfataricus,* the CRISPR-Cas system is able to trigger immunity even when several mismatches between crRNA and its target sequence are present, indicating that the archaeal system might be more promiscuous than the currently studied bacterial systems.

To compare the archaeal system with other bacterial CRISPR systems and to dissect potential differences, we investigate here the minimal requirement of sequence similarity between crRNAs and protospacers in *S. solfataricus,* and analyse if a potential SEED sequence can be dissected. We explore the basis for self-/non–self-discrimination by crRNAs, and we also shed light on the need of a PAM sequence in the interference process.

## MATERIALS AND METHODS

### Strain growth and plaque assay

Strains *S. solfataricus* P1 and P2 (DSM 1616, 1617) were grown at pH 3 and 78°C in Brock’s medium (24) using long-necked flasks, with 0.1% (w/v) tryptone and 0.2% sucrose, in a shaking incubator. The optical density of liquid cultures was monitored at 600 nm. Plates were prepared by adding gellan gum (Gelrite; Kelco Biopolymers) to the Brock’s solution to a final concentration of 0.6% and Mg^2+^ and Ca^2+^ to 0.3 and 0.1 M, respectively. For the plaque assays, 5 µl of transfected cells were mixed with 300 µl of 10× concentrated, logarithmically grown cells (for the lawn) and with 2 ml of pre-warmed growth medium supplemented with 0.3% gellan gum (final concentrations) without carbon source. The mixture was quickly poured on plates. Plaques formed after incubation for 2 days at 78°C.

### Preparation of vector constructs

The ORF 406 of pNOB8 plasmid (25) was amplified with primers 406Fw and 406Rw using Phusion DNA polymerase (Finnzymes) with the following polymerase chain reaction (PCR) condition: 96°C for 2 min, 30 cycles of 96°C 15 s, 52°C 20 s, 72°C 1 min 30 s followed by 72°C 5 min. The PCR product was gel purified (kit) and cloned into the pCR8-GW cloning vector (Invitrogen). Plasmid DNA was extracted (E.Z.N.A. Plasmid Miniprep KitI, Omega Bio-tek) and 1 ng of plasmid was used as a template for inverse PCR with mutagenic primers (see Supplementary Table S1 for primer sequences). PCR was performed in 1× GC Buffer, 1 U of Phusion polymerase 0.4 mM dNTPs, and 400 pmol of mutagenesis primer mix using the following PCR conditions: 96°C 2 min, 33 cycles of 96°C 15 s, 58°C 15 s, 72° 3 min with a final extension of 5 min at 72°C. Primers were phosphorylated before use, mixing 2 µl of 100 µM FW and RW mutagenic primer with 2 µl of T4 kinase buffer and 1 U of T4 DNA kinase in a 20-µl reaction volume and incubating for 1 h at 37°C. In Supplementary Table S1, the different primer combinations for each construct are listed. The linear plasmids amplified by inverse PCR were cleaned using a PCR clean-up kit (EZNA) and digested with 2 U of *Dpn*I (to digest the non-amplify wild-type plasmid) for 2 h at 37°C. After digestion, the plasmids were column purified, and 50 ng was used in a ligation reaction with 1 U of T4 Ligase (New England Biolabs) and 2 µl of 10× T4 ligase buffer in a volume of 50 µl and incubation for 3 h at 21°C. Two microliters of re-circularized plasmids was then used to transform TOP10® cells (Invitrogen). Positive colonies were screened by PCR, and the mutated protospacer sequence was analysed by Sanger sequencing.

Correct clones were amplified and purified by plasmid preparation, and 100 ng of the DNA was used in a gateway LR-recombination reaction according to the manufacturer’s instructions (Invitrogen), with the pDEST gateway vector pMJ-GW (see Supplementary Figure S1). This vector was designed by placing the gateway cassette A (Invitrogen) between the restriction sites AvrII and EagI of the *Sulfolobus* shuttle vector pMJ03-05 (26).

### Transfection assay and plaque-forming unit normalization

DNA of the recombinant shuttle vectors was extracted from transformed *E. coli* cells using E.Z.N.A. Plasmid Miniprep KitI Omega Bio-tek and quantified in a Nano-Drop spectrometer (PeqLab); then an aliquot was loaded on an 0.8% agarose gel to analyse its purity and topology. In all experiments 150 ng of viral DNA was used to transfect electrocompetent *Sulfolobus* cells (27) using 1-mm cuvettes (PeqLab) with the following electroporation condition: 1250 V/25 vµF/1000 vΩ in a gene pulser (Biorad). After transfection, the cells were incubated for 1 h in recovery solution (28) at 75°C. The transfected cells were then used for plating (plaque assay). Transfection efficiencies were determined in triplicate by counting plaque-forming units (PFU) on plates and normalizing to the viral vector with the highest plaque count.

## RESULTS

### Minimal requirement of sequence complementarity between crRNA and protospacer

In our previous work, we have demonstrated that the CRISPR system in *S. solfataricus* can recognize and trigger degradation of a protospacer also when up to four mutations are introduced into the protospacer region, and that the efficiency of transfection decreased with the increase of the number of mismatches between the spacer and protospacer (22).

**Figure 1.**
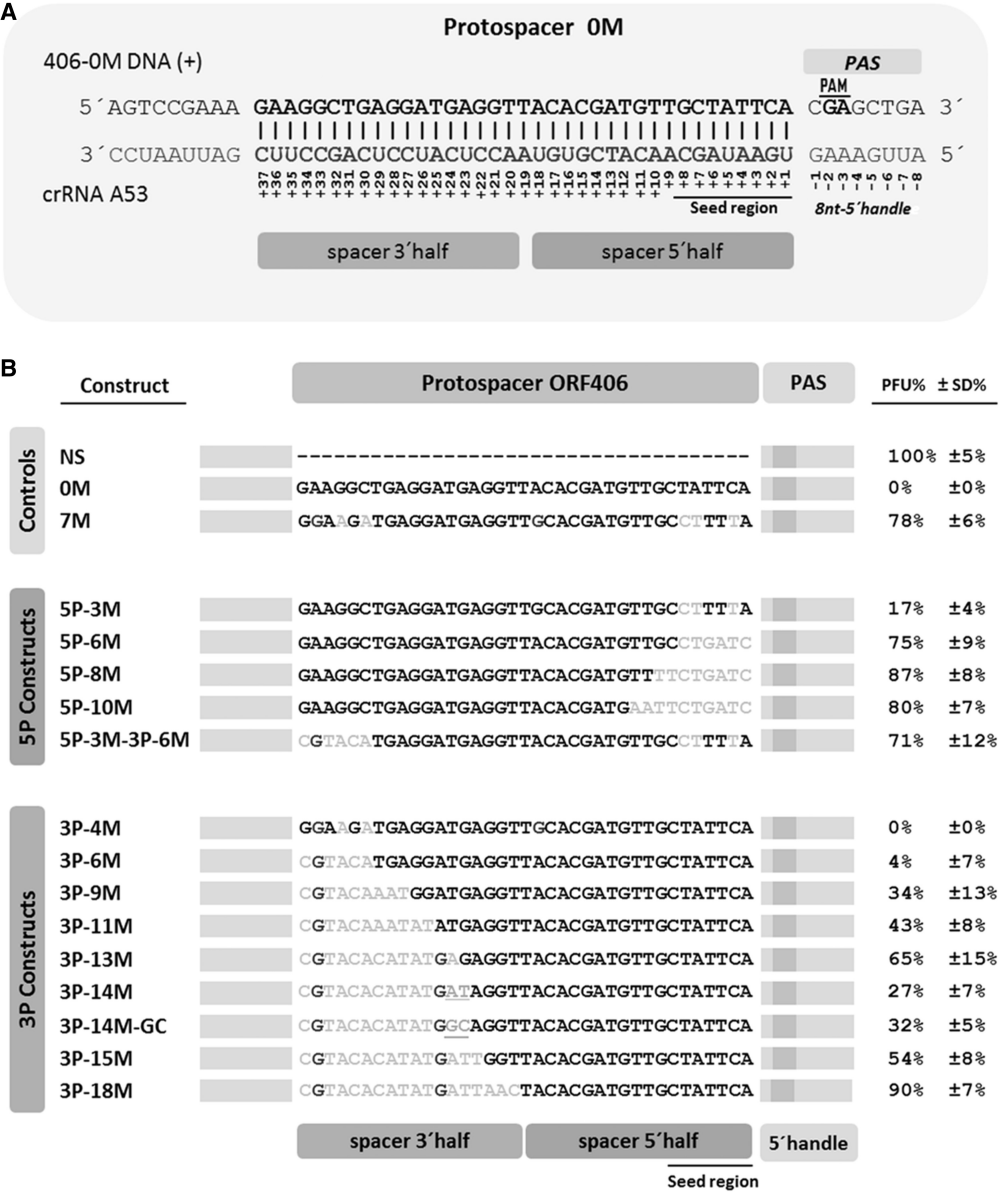
(**A**) Schematic representation of the crRNA-protospacer interaction region, as well as flanking sequences, including 5′ handle, PAM and PAS. (**B**) CRISPR-mediated DNA interference as measured by the capability of plaque formation of different protospacer-containing virus DNA constructs. Controls were NS, which did not carry the protospacer sequence (negative control), 0M, which carries a protospacer sequence in the ORF406 homologous to the crRNA53 of CR3 (positive control) and 7 M, the wild-type pNOB ORF406 sequence with 7 mutations to the crRNA. 5-P and 3-P distinguish the constructs with respect to the location of mutations in the 5′ half and 3′ half, respectively, and further numbers indicate the number of mutations between crRNA and protospacer. Transfection efficiencies of each construct are given as percentage of that of the control construct NS. Bars represent standard deviations of ≥3 replicates.

To understand the minimal requirement of mutations that can completely abolish the DNA interference, we constructed 14 new viral variants carrying a mutated protospacer and used those to transfect *S. solfataricus*. Each virus variant was designed to carry the ORF 406 of the conjugative plasmid pNOB8 (25) in which the protospacer sequence matching spacer n°53 of CRISPR locus A was mutated to a different degree (Figure 1A). Each viral DNA was successively analysed for its ability to persist in *S. solfataricus* strain P2, and the transfection rate was quantified in a plaque assay.

The viral vectors were divided into two groups: the ‘5P’ constructs and the ‘3P’ constructs (see Figure 1B). The ‘5P’ constructs contained mismatches between the 5′ half of the spacer and the protospacer, whereas the ‘3 P’ constructs were designed to study the importance of the interaction between the 3′ half of the spacer and the targeted sequence (see Figure 1B). Whereas construct 5P–3M (three mismatches with respect to the spacer) had a transfection efficiency of ∼20% compared with the control vector NS (no interference) carrying the full ORF406 without any protospacer, transfection efficiency rose to 75% for construct 5P–6M, and even to ∼85% for 5P–8M and 5P–10M. These results demonstrate that more than six mutations in the interaction region between the 3′-end of the protospacer and the 5′ half of the spacer strongly affect and eight and more mutations almost completely abolish the DNA interference in *S. solfataricus*.

A different outcome was obtained when testing the region of interaction on the other side, i.e. between the protospacer and the spacer 3′ half. Seven different protospacer sequences with different numbers of mutations (Figure 1B) were cloned into the viral vector, with 6 (3P–6M), 9 (3P–9M), 11 (3P–11M), 13 (3P–13M), 14 (3P–14M), 15 (3P–15M) and 18 (3P–18M) nt being replaced at the 5′-end of the 0M protospacer. In addition to the above constructs, vector 3P–4M also was tested, as previously done (22), which carries four mutations in positions +36, +34, +32 and +18 (in positions 36 and 18 G–U pairing between the spacer and protospacer was possible in the DNA–RNA hybrid). The transfection efficiency of the constructs with respect to the control is reported in Figure 1B. Four mutations (3P–4M) showed no effect on DNA degradation. The construct was still cleaved to the same degree as the perfect matching protospacer 0M. Six consecutive mutations of 3P–6M increased the ability to form plaques (PFU) only to ∼5%, and only nine mutations increased the efficiency of transfection to ∼35%. Eleven and 13 mutations were still not sufficient to fully abolish DNA degradation; in fact, the viral vectors 3P–11M and 3P–13M caused only ∼45 and ∼65% of plaques when electroporated into *Sulfolobus* cells, respectively. Surprisingly, plaque formation dropped again from ∼65% to ∼30% with construct 3P–14M, whereas construct 3P–15M formed ∼55% of plaques compared with the control NS vector. The transfection efficiency rose to that of the control (NS) only when the viral vector 3P–18M was tested (>90%). To understand whether the difference in transfection efficiency of the construct 3P–13M and 3P–14M was due to reasons not related to sequence complementarity but rather e.g. to plasmid quality, we used the same constructs to transfect *S. solfataricus* strain P1, a close relative of strain P2 that does not contain spacer A53 in its genome. No significant differences in infectivity were found demonstrating that the transfection capacity of all viral vectors was equally high.

Because the different transfection efficiencies of the viral vectors 3P–13M and 3P–14M could not be explained by differences in plasmid quality, we investigated the possibility that the replaced nucleotides in the mutated protospacer sequences could be directly involved in lowering or increasing the CRISPR activity. We therefore focused our attention on the single nucleotide difference between constructs 3P–13M and 3P–14M. In construct 3P–14M, a single G base had been mutated to a T base to increase the number of mismatches between the spacer and its target by one nucleotide. This mutation had created an AT dinucleotide sequence immediately in front of the region of homology between protospacer and spacer (Figure 1B). To understand whether this AT dinucleotide could be a putative CRISPR cutting site [as previously seen for the *S. solfataricus* Cmr complex (29)], which could increase protospacer degradation in construct 3P–14M, we replaced the AT sequence in position 24–25 with the dinucleotide GC. Interestingly, the transfection efficiency of the newly designed vector was ∼30%, as for construct 3P–14M. Thus, the reason of this different transfection ability between vectors 3P–13M and 3P–14M remained obscure. We also excluded the possibility that RNA secondary structure could be a trigger for higher infectivity of the viral vector 3P–13M because the ORF406 is not transcribed in our viral vector and we are directly manipulating the target DNA and not the spacer A53.

### Synergistic effect of mutations

The above results indicate that the ability of the different viral vectors to escape the CRISPR system is mostly proportional to the amount of mutations and that every mutation has a different ‘weight’ depending on its localization along the interaction region between protospacer sequence and crRNA. Mutations toward the 5′-end of the protospacer (=3′ half of spacer) do not or only minimally influence the ability of the virus to escape the CRISPR defence (see Figure 1B constructs 3P–4M, 3P–6Md, 3P–9Md). However, we have noticed in our earlier study that these less important positions also influence interference when they occur in combination with others. In (21), constructs 406–7M and 406–3M (here reported as 7M and 5P–3M) had been tested. The constructs 5P–3M and 3P–4M (see above) are derivatives of construct 7M, where the mutations at the 5′ half (5P–3M) and 3′ half (3P–4M) of the 7M protospacer, respectively, were replaced to perfectly match the spacer A53. The transfection efficiency described for construct 5P–3M was ∼20% and for construct 3P–4M was ∼0%. Interestingly, construct 7M, which had both sets of mutations (5P–3M and 3P–4M), escaped the CRISPR system in ∼80% of the cases. These data show that those additional mutations at the opposite side of the protospacer triggered a synergistic effect, increasing the capacity of the virus to escape the CRISPR system. To confirm this result, we designed a new protospacer called 5P–3M–3P–6M, in which the mutations of construct 5P–3M were combined with those of the 3P–6M construct. The PFUs obtained with construct 5P–3M–3P–6M (∼70%) were higher than those of both parental constructs: 5P–3M (∼20%) and 3P–6M (∼5%). These results indicate that base pairing in the first six nucleotides of the 3′ half of the crRNA are also important for the interference process, as mutations decrease the CRISPR system efficiency when occurring together with mutations within the seed sequence of crRNA.

### Self-/non–self-target recognition in *S. solfataricus*

In *S. epidermidis*, the presence of at least three complementary nucleotides in the 3′ flanking region of the PAS is needed for ‘self-recognition’, i.e. for protecting the protospacer from degradation and thus to avoid the targeting of the CRISPR locus by the self-originated spacers (17).

**Figure 2.**
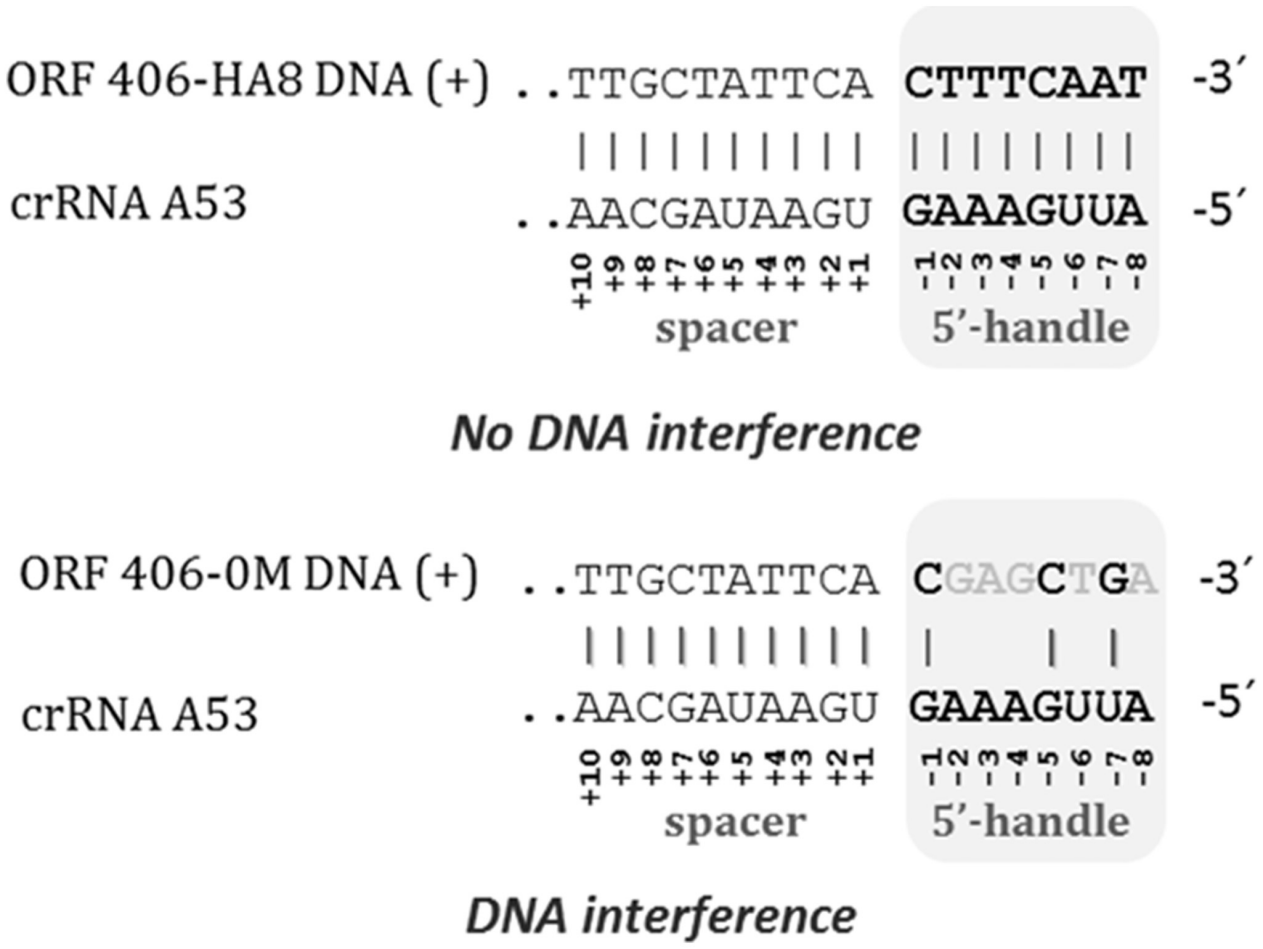
Protospacer adjacent sequence (PAS) of ORF406 (positions −1 to −8) and the 8-nt 5′ handle of the crRNA A53 of CR3 in constructs HA8 (**A**) and 0 M (**B**).

To increase the understanding of this process in archaea, we mutated the PAS of the protospacer 0M on the viral vector pMJ to match the 5′ handle of the corresponding crRNA, which stems from the last 8 nt of the repeat present in the CRISPR locus (CRISPR A-B of *S. solfataricus*) (Figure 2)*.*

**Figure 3.**
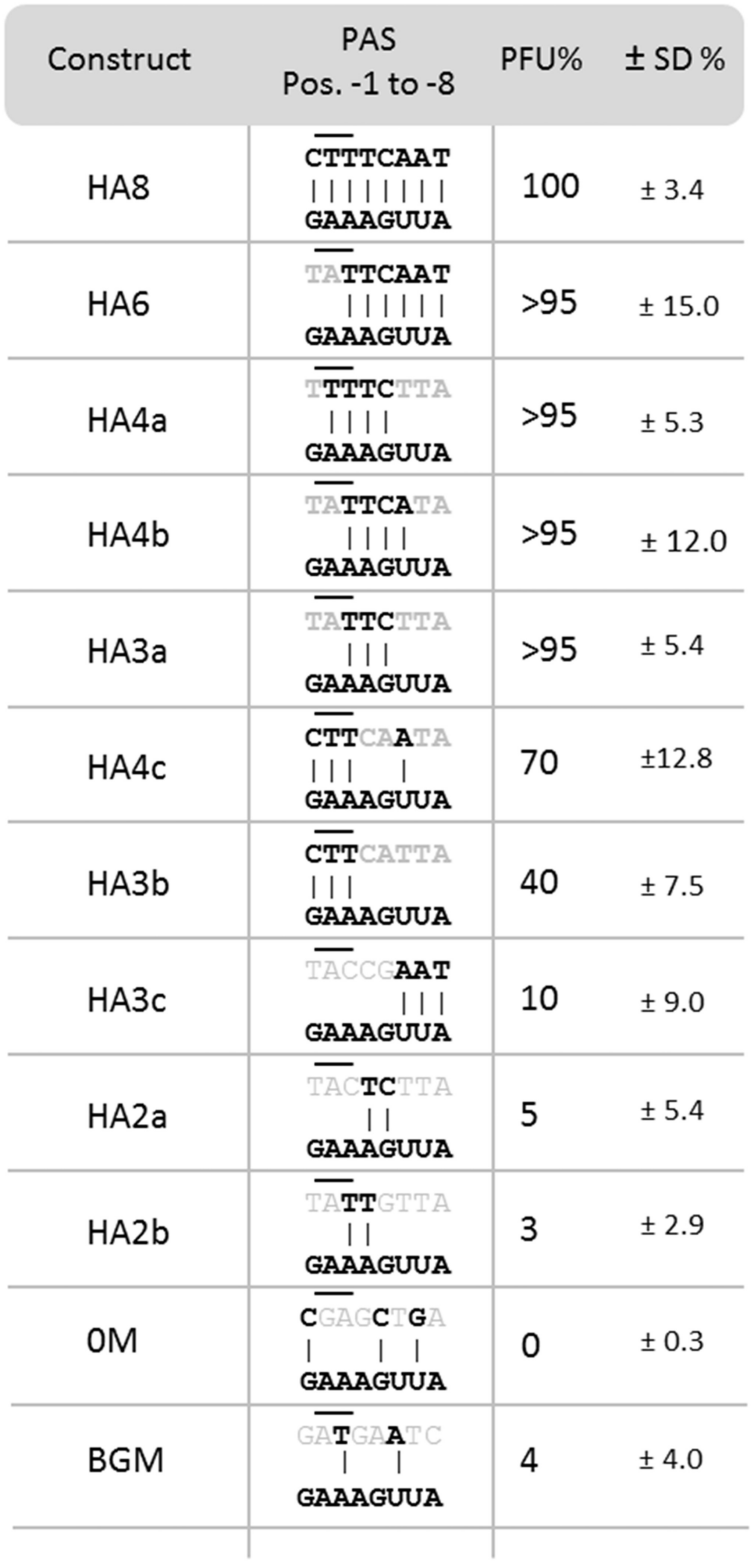
Self- versus non–self-target discrimination. Numbers in constructs (first column) refer to the number of matching nucleotides between PAS and crRNA’s 5′ handle. In column 2, the similarity between PAS and 5′ handle is shown, and in column 3 the plaque formation of the different constructs on transfection is given (with respect to the 100% control HA8). At least three consecutive nucleotide matches in positions −3, −4, and −5 were necessary to block interference. The nucleotide sequences of positions −2, and −3 (putative PAM position) of each construct is shown with a black line above the corresponding nucleotides. The different constructs are reported in the order of their ability to escape the CRISPR system. The transfection efficiency of the different constructs is reported in column 4 with the respective standard deviation of ≥3 replicates.

The results are reported in Figure 3. Construct HA8 (HA for HAndle), in which the PAS of the protospacer matched perfectly the 8-nt-long 5′ handle of the crRNA, was no more a target of the CRISPR-mediated DNA interference, i.e. yielded as many plaques as a virus without a protospacer.

We then reduced the number of matches between the flanking sequence and 8-nt 5′ handle to characterize the minimal requirement for full protection. Six-nucleotide complementarity (HA6) and even only 4 nt (HA4a and HA4b) fully protected the target, when the protospacer had at least three consecutive matches with the 5′ handle between positions −3 and −5 (see Figure 3). Four matches, when covering different positions in the 5′ handle (positions −1 to −3 and position −6), did not confer full protection but partially protected the protospacer from degradation (30% for HA4c). To identify which positions in the flanking sequence are important for the protection, we then positioned three consecutive matching nucleotides at different places in the PAS (HA3a, HA3b and HA3c) (Figure 3). Construct HA3c, which carried three consecutive nucleotides complementary to the last three nucleotides of the 5′ handle (positions −6, −7 and −8), conferred only ∼10% of immunity, and construct HA3b with three nucleotides matching the first three positions of the 5′ handle (positions −1, −2 and −3) conferred ∼40% with respect to the control 0M construct. Full immunity was obtained, with construct HA3a having the three consecutive nucleotide matches at the positions −3, −4 and −5 between PAS and the 5′ handle.

The plaque-forming ability of two constructs with only two matching nucleotides in the same critical region (HA2a and HA2b) was ∼5%, indicating that at least three matches between 5′-handle and PAS are necessary to achieve self- from non-self-discrimination in archaea.

To verify whether the self-/non–self-recognition process needs a full match between protospacer and crRNA to be active, we designed three new viral vectors called 3P-6M-HA, 3P-14M-HA and 5P-3M-HA. Those vectors carried the protospacer 3P–6M, 3P–14M and 5P–3M, respectively, while their PAS was mutated to match perfectly the 5′ handle. All three constructs independently of the amount of mutations and their positions along the protospacer were fully protected from degradation having a >95% capability of plaques formation compared with the control virus 0M (∼0%).

These results demonstrate that the presence of a perfectly matching PAS sequence blocks CRISPR-mediated DNA degradation also of protospacers that do not have 100% homology to the respective crRNA.

### PAM sequence and DNA interference

To elucidate the importance of the PAM in *Sulfolobus*, we used the above constructs and their protospacer flanking sequence. As described above, the construct 0M carries a 37-nt-long protospacer perfectly matching spacer A53. Its upstream flanking sequence contains a previously described *S. solfataricus* PAM motif (GA) (3), and this construct is fully recognized and cleaved by the CRISPR system under our test conditions.

To analyse whether the DNA interference activity is affected by the presence of the PAM dinucleotide sequence, we introduced the same 37-nt protospacer into a different genetic background (position +283/+320 of SSO3019 lacS). The new viral vector BGM showed the dinucleotide sequence AT in positions −2 and −3. Only 5% difference in transfection efficiency was noticed between the constructs 0M and BGM (Figure 3). Both constructs were unable to efficiently infect *S. solfataricus* P2 cells bearing an active CRISPR locus demonstrating that the DNA interference activity of the CRISPR system is independent of a PAM sequence. Furthermore, constructs HA4c and HA3b (Figure 3) that were used to demonstrate the self-/non–self-recognition carry the nucleotide sequence TT in positions −2 and −3; both triggered DNA interference in *S. solfataricus* P2 and were able to escape the CRISPR system in ∼70 and ∼40% of the cases, respectively (Figure 3). Both viral vectors should have had the same transfection efficiency, if the reason for escape of these virions were related to the absence of a PAM sequence. As already mentioned, the difference in transfection efficiency found between these two constructs can be explained by the degree of similarity between PAS (protospacer flanking sequence) and 5′ handle of the spacer. These results were also confirmed by the transfection efficiency of constructs HA2a and HA2b, which carry the dinucleodite sequence AC and AT in the PAM positions −2, and −3, and which are nevertheless recognized and cleaved by the CRISPR system.

## DISCUSSION

### High permissiveness of the CRISPR activity in *S. solfataricus*

Our study demonstrates the extraordinary ability of the *S. solfataricus* CRISPR-Cas system in targeting protospacers with high numbers of mismatches to the complementary crRNA. Even up to 15 mismatches between protospacer and the 3′ half of the crRNA can be tolerated by the system and still trigger 50% of DNA degradation. However, similar to the findings in *E. coli* (18), also in *S. solfataricus,* mutations between positions +1 and +8 (‘seed’ sequence) determine a rapid decrement in the interference process. In our system, viruses carrying protospacers with three mutations in the seed sequence are able to escape in 20% of the cases. The DNA interference process is almost completely abolished when more than six mutations between spacer and protospacer are present in the first 8 nt of the 5′ half region of the spacer. We conclude that the first 6–8 nt of the spacer are of crucial importance in target recognition also in archaea, and that the seed sequence might be a characteristic of all CRISPR systems, or at least of type I systems. In archaea, but maybe also in many bacteria, the system is far less stringent with respect to mutations on the opposite side of the spacer. However, we found that mutations within this (less important) protospacer region can enhance protection when mutations in the seed sequence occur in addition, thus triggering a synergistic effect. This might explain (in parts) why the length of the spacers is maintained during evolution of the CRISPR systems. Future experiments on the interference mechanism will be needed to fully understand this synergistic effect.

It will be interesting to elucidate whether a CRISPR system can eradicate a virus from a population when it is targeted with partially matching crRNAs or whether this virus will be maintained at lower levels, e.g. intracellularly at lower copy numbers. If the latter is true, the view of the CRISPR system in virus defence should be extended from a mere ‘first barrier defence mechanism’ to a ‘virus defence and maintenance’ system. The ability to maintain viruses at lower levels might be beneficial permitting invasion of genetic elements that carry genes that are favourable to the host. Those viruses would probably get slowly lost if the host does not gain a selective advantage, but would be kept at low levels as long as they are beneficial.

Furthermore, maintaining a virus alive and controlling its copy number through the CRISPR system, thus avoiding excessive replication and a lytic cycle might be an effective mechanism of defence as well. Recently, Swarts *et al.* demonstrated that the presence of CRISPR spacers matching the invading genetic element seems to facilitate the incorporation of additional spacers (30). In the light of this finding, the presence of spacers targeting an invading genetic element (even with low similarity) would allow the system to readily respond against virus over-proliferation. On infection, the virus titre could reach a threshold at which the concentration of partially matching spacer and its target would allow pairing and a first (low efficiency) degradation of the target. This degradation of the protospacer could then induce the acquisition of new spacers, now perfectly matching the viral DNA. The newly acquired spacers will then block the viral proliferation allowing the CRISPR interference cassettes to target the invader with high efficiency and to eliminate it (30). Thus, high permissibility (i.e. tolerance of mutations between spacers and protospacers) would represent an effective system against a huge diversity of genetic elements as found in *Sulfolobales* (31).

It will be interesting to see whether the extraordinary ability of the *Sulfolobus* CRISPR-Cas system to target highly diverse protospacers is also found in other bacteria or archaea or whether it remains a special feature of *Sulfolobus* or hyperthermophiles in general. We cannot rule out the possibility that the high tolerance of mutations could be specific for these organisms, because RNA–DNA hybrids do not easily form at high temperatures and might have to be specifically supported through the action of protein complexes.

### Autoimmunity

Despite the huge variety of CRISPR-Cas systems found in different organisms, the match between 8-nt-long 5′ handle of the crRNA and PAS seems to be a common trade to avoid self-degradation. Indeed, in *Sulfolobus*, the complete match between 5′ handle and PAS protospacer flanking sequence blocked the interference process completely and a high degree of protection was still maintained even with only three matches in positions −3, −4, and −5. Also in bacteria (although at different positions), three matches between PAS and crRNA 5′ handle were the minimal requirement to protect the CRISPR locus from its own degradation (17). Our results are also in general compatible with the recent finding in *Sulfolobus islandicus* REY15A where similarity between crRNA 5′ handle and PAS sequence conferred protection against CRISPR DNA targeting, although different from that study, we found only 40% of protection when homology between positions −1 and −3 was present (32).

### Role of the PAM sequence

We have also evaluated the need of a PAM sequence for triggering DNA interference. Our results indicate that it is not necessary for triggering DNA interference, at least not for the spacer of CRISPR locus A analysed in our study. Our data strongly support the hypothesis that mutations in the PAM region decrease transfection efficiency only when they increase the similarity between the crRNA 5′ handle and PAS, triggering self-/non–self-discrimination. Previous results in *S. solfataricus* and *S. islandicus* (3) have demonstrated the need of a PAM sequence to trigger DNA interference. These different results could be explained by the use of different protospacers targeted by crRNAs of different CRISPR loci sub-families. *S. solfataricus* carries seven different CRISPR-Cas systems with three different repeat types and three putative CASCADE cassettes, a Csm and a Cmr module (9,33,34). So far, only the *in vitro* targeting activity of one Cmr complex was elucidated (29). Interestingly, Lintner *et al.* reported that no alteration in binding affinity between *Sulfolobus* CASCADE-crRNA complex and protospacer was found in the presence or absence of a PAM motif, contrarily to what was found in other systems (16). No *in vitro* DNA targeting activity has been tested yet for the CASCADE complex studied in this work. Furthermore, it should be mentioned that the crRNAs that co-purify together with one CASCADE module (16) originate from different CRISPR loci. This underlines the possibility that different spacers could associate with different CRISPR interference complexes within the cell, which might have different requirements with respect to the PAM site. Whether the different interference complexes discriminate the different spacers is still under debate, but it is likely that the crRNA studied here could be recognized by a CRISPR interference complex different from the one used in Gudbergsdottir experiments (3). Indeed, *S. solfataricus* possesses a CRISPR type III-A system (Csm module), which was found in bacteria to be insensitive to the presence of a PAM site for triggering its DNA degradation activity (17).

Another possible explanation for the different outcome of the two experiments rises from the different approaches used to determine CRISPR activity. In our assay, an engineered virus carrying a matching protospacer is transfected into the cells and needs to proliferate and spread to the neighbouring cells to trigger plaque formation. This approach differs from the plasmid-based approach used in the previous studies to determine PAM function. It is possible that the presence of our vector inside the cell increases Cas expression determining higher interference activity, which would perhaps not require a PAM site.

In a mutational study of *Haloferax volcanii*, Fischer *et al.* have shown that six different variants (trinucleotides) can function as a PAM when the organism is challenged with a plasmid (35).We cannot completely rule out that in our system also there is a requirement of a PAM for target recognition, but that we do not identify it because many variants of it can be tolerated. However, we consider it unlikely as it would mean that we have by chance picked the right nucleotides when exchanging the existing potential ‘PAM’ from a GA (in construct 0M) to an AT (in BGM and HA2b) or an AC (HA2a), respectively.

More work will be needed to define the role of sequences lying adjacent to the protospacer in the invader and to distinguish it in the different CRISPR types. The sequence requirements of the PAM motif might also be different for the two different functional roles (acquisition and interference). Therefore, it has recently been suggested to differentiate between a spacer acquisition motif (SAM) and a target interference motif (TIM) (36). In light of this, our results indicate that a target interference motif does not seem to play a role in target interference of our system.

## SUPPLEMENTARY DATA

Supplementary Data are available at NAR Online.

## FUNDING

The SulfoSYS-project [SysMo P–N-01-09-23] and by FWF project [P23000 to C.S.]. Funding for open access charge: University of Vienna.

*Conflict of interest statement*. None declared.

## Supplementary Material

Supplementary Data
