# Supplementary material for: Unexpectedly broad target recognition of the CRISPR-mediated virus defence system in the archaeon Sulfolobus solfataricus
Source: Nucleic Acids Res. 2013 Sep 9;41(22):10509–17. doi: 10.1093/nar/gkt767 (PMC3905844; doi:10.1093/nar/gkt767)
Supplement: Supplementary Data [file supp_41_22_10509__index.html]

Unexpectedly broad target recognition of the CRISPR-mediated virus defence system in the archaeon Sulfolobus solfataricus — Unexpectedly broad target recognition of the CRISPR-mediated virus defence system in the archaeon Sulfolobus solfataricus — Supplementary Data 

# Unexpectedly broad target recognition of the CRISPR-mediated virus defence system in the archaeon *Sulfolobus solfataricus*

## Supplementary Data

files

**Files in this Data Supplement:**

- Supplementary Data - pdf file
