# Supplementary material for: Unexpectedly broad target recognition of the CRISPR-mediated virus defence system in the archaeon Sulfolobus solfataricus
Source: Nucleic Acids Res. 2013 Sep 9;41(22):10509–17. doi: 10.1093/nar/gkt767 (PMC3905844; doi:10.1093/nar/gkt767)
Supplement: Supplementary Data [file supp_gkt767_nar-01422-h-2013-File005.pdf]

SUPPLEMENTARY DATA

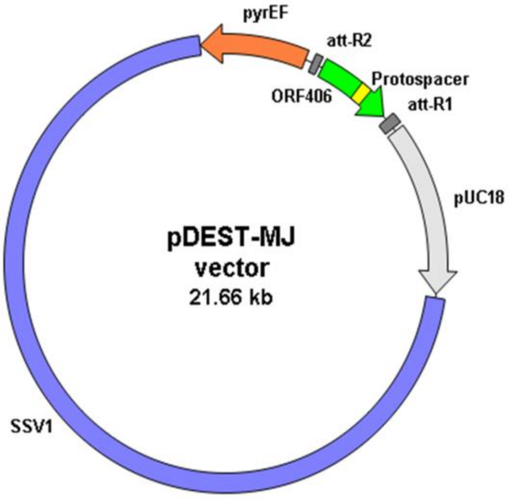

Fig.S1: Gateway destination vector pDEST-MJ, i.e. shuttle vector between *E. coli* and *S. solfataricus* based on the Sulfolobus SSV1 virus and pUC18.

DNA interference constructs				
TEMPLATE NAME	FRAGMENT NAME	PRIMER NAME	PRIMER SEQUENCE 5'-> 3'	TARGET SEQUENCE
pCR8-0M	5P-6M	Rw1-406-6M-up	GCAACATCGTGTAACCTCATCC	GTCCGAAA**GAAGGCTGAGGATGAGGTTACACGATGTTGCCTGATC**CGAGCTGA
		Fw1-406-6M-over-up	CTGATCCGAGCTGAAAAGCATCTTGAAG	
pCR8- 5P-6M	5P-8M	Fw-8M-UP	GATCAGAAAACATCGTGTAACCTC	GTCCGAAA**GAAGGCTGAGGATGAGGTTACACGATGTTTTCTGATC**CGAGCTGA
		Fw-WOP	CGAGCTGAAAAGCATCTTGAA	
pCR8- 5P-6M	5P-10M	Fw-10M-up	GATCAGAATTCATCGTGTAACCTC	GTCCGAAA**GAAGGCTGAGGATGAGGTTACACGATGAATTCTGATC**CGAGCTGA
		Fw-WOP	CGAGCTGAAAAGCATCTTGAA	
pCR8-0M	3P-6M	Fw2-406-6M-down	TGAGGATGAGGTTACACGATGTT	GTCCGAAA**CGTACATGAGGATGAGGTTACACGATGTTGCTATTCA**CGAGCTGA
		Rw2-406-6M-over-down	GTACGTTTCGGACTTTTCCACCAACT	
pCR8-3P-6M	3P-9M	Rw-9M-down	CGTACAAATGGATGAGGTTACAC	GTCCGAAA**CGTACAAATGGATGAGGTTACACGATGTTGCTATTCA**CGAGCTGA
		Rw-WOP	TTTCGGACTTTTCCACCAACT	
pCR8-3P-13M	3P-11M	Fw4-406-13M-down	GAGGTTACACGATGTTGCTATTCA	GTCCGAAA**CGTACACATATATGAGGTTACACGATGTTGCTATTCA**CGAGCTGA
		Rw-13M(+at)	ATATATGTGTACGTTTCGGACTTTT	
pCR-3P-14M	3P-13M	Fw4-406-13M-down	GAGGTTACACGATGTTGCTATTCA	GTCCGAAA**CGTACACATATGAGAGGTTACACGATGTTGCTATTCA**CGAGCTGA
		Rw4-406-13M-specific	TCATATGTGTACGTTTCGGACTT	
pCR8-0M	3P-14M	Fw3-406-14M-down	AGGTTACACGATGTTGCTATTCAC	GTCCGAAA**CGTACACATATGATAGGTTACACGATGTTGCTATTCA**CGAGCTGA
		Rw3-406-14M-over-down	ATCATATGTGTACGTTTCGGACTTTTCCACCAACT	
pCR-3P-14M	3P-14M-GC	Fw3-406-14M-down	AGGTTACACGATGTTGCTATTCAC	GTCCGAAA**CGTACACATATGGCAGGTTACACGATGTTGCTATTCA**CGAGCTGA
		Rw-14M(-at)	GCCATATGTGTACGTTTCGGACT	
pCR-3P-14M	3P-15M	Fw5-406-15M-down	TTGGTTACACGATGTTGCTATTC	GTCCGAAA**CGTACACATATGATTGGTTACACGATGTTGCTATTCA**CGAGCTGA
		Rw4-406-13M-specific	TCATATGTGTACGTTTCGGACTT	
pCR8-3P-15M	3P-18M	Fw-18-down	CGTACACATATGATTAACTACACGATGT	GTCCGAAA**CGTACACATATGATTAACTACACGATGTTGCTATTCA**CGAGCTGA
		Rw-WOP	TTTCGGACTTTTCCACCAACT	
pCR8- 5P-6M	5P-3M-3P-6M	Fw-WOP	CGAGCTGAAAAGCATCTTGAA	GTCCGAAA**CGTACATGAGGATGAGGTTACACGATGTTGCCTTTTA**CGAGCTGA
		Rw_3Mup	TAAAAGGCAACATCGTGTAACC	
pCR8-0M	NS	Fw-WOP	CGAGCTGAAAAGCATCTTGAA	GTCCGAAA-------------------------------------CGAGCTGA
		Rw-WOP	TTTCGGACTTTTCCACCAACT	
pSVA5	BGM	BGM406A_RW	CCTCATCCTCAGCCTTCCCTTGGTAATGGATTAGGAAATA	TACCAAGG**GAAGGCTGAGGATGAGGTTACACGATGTTGCCTTTTA**GATGAATC
		BGM406A_FW	TTACACGATGTTGCTATTCAGATGAATCAAAACAAGATGTGAC	

Tab.S1: List of all primers and template plasmids used for the preparation of the different protospacer constructs for the DNA interference experiments. In the first two columns the name of the inverse PCR template plasmid and the name of the final construct are given. In the third column the primer name followed by the primer sequence in column 4. The sequence 5´-> 3´ of the different protospacers is reported in column 5. In bold the nucleotides which correspond to the protospacer sequence, underlined the 8 nucleotides of the protospacer adjacent sequence.

Self non-self discrimination constructs				
TEMPLATE NAME	FRAGMENT NAME	PRIMER NAME	PRIMER SEQUENCE 5'-> 3'	TARGET SEQUENCE
pCR8-0M	HA8	Rw1	TGAATAGCAACATCGTGTAACCT	GTCCGAAA**GAAGGCTGAGGATGAGGTTACACGATGTTGCTATTCA**CTTTCAAT
		Fw1	CTTTCAATCACCCTTAGTGCCTGTGGAG	
pCR8-0M	HA6	Rw1	TGAATAGCAACATCGTGTAACCT	GTCCGAAA**GAAGGCTGAGGATGAGGTTACACGATGTTGCTATTCA**TATTCAAT
		Fw5	TATTCAATCACCCTTAGTGCCTGTGGAG	
pCR8-0M	HA4a	Rw1	TGAATAGCAACATCGTGTAACCT	GTCCGAAA**GAAGGCTGAGGATGAGGTTACACGATGTTGCTATTCA**TTTTCTTA
		Fw6	TTTTCTTACACCCTTAGTGCCTGTGGAG	
pCR8-0M	HA4b	Rw1	TGAATAGCAACATCGTGTAACCT	GTCCGAAA**GAAGGCTGAGGATGAGGTTACACGATGTTGCTATTCA**TATTCATA
		Fw9	TATTCATACACCCTTAGTGCCTGTGGAG	
pCR8-0M	HA4c	Rw1	TGAATAGCAACATCGTGTAACCT	GTCCGAAA**GAAGGCTGAGGATGAGGTTACACGATGTTGCTATTCA**CTTCAATC
		Fw2	CTTCATTACACCCTTAGTGCCTGTGGAG	
pCR8-0M	HA3a	Rw1	TGAATAGCAACATCGTGTAACCT	GTCCGAAA**GAAGGCTGAGGATGAGGTTACACGATGTTGCTATTCA**TATTCTTA
		Fw7	TATTCTTACACCCTTAGTGCCTGTGGAG	
pCR8-0M	HA3b	Rw1	TGAATAGCAACATCGTGTAACCT	GTCCGAAA**GAAGGCTGAGGATGAGGTTACACGATGTTGCTATTCA**CTTCATTA
		Fw2	CTTCATTACACCCTTAGTGCCTGTGGAG	
pCR8-0M	HA3c	Rw1	TGAATAGCAACATCGTGTAACCT	GTCCGAAA**GAAGGCTGAGGATGAGGTTACACGATGTTGCTATTCA**TACCGAAT
		Fw3	TACCGAATCACCCTTAGTGCCTGTGGAG	
pCR8-0M	HA2a	Rw1	TGAATAGCAACATCGTGTAACCT	GTCCGAAA**GAAGGCTGAGGATGAGGTTACACGATGTTGCTATTCA**TACTCTTA
		Fw-F12	TACTCTTACACCCTTAGTGCCTGTGGAG	
pCR8-0M	HA2b	Rw1	TGAATAGCAACATCGTGTAACCT	GTCCGAAA**GAAGGCTGAGGATGAGGTTACACGATGTTGCTATTCA**TATTGTTA
		Fw-F14	TATTGTTACACCCTTAGTGCCTGTGGAG	
pCR8- 3P-6M	3P-6M-F8	Rw1	TGAATAGCAACATCGTGTAACCT	GTCCGAAA**CGTACATGAGGATGAGGTTACACGATGTTGCTATTCA**CTTTCAAT
		Fw1	CTTTCAATCACCCTTAGTGCCTGTGGAG	
pCR8-3P-14M	3P-14M-F8	Rw1	TGAATAGCAACATCGTGTAACCT	GTCCGAAA**CGTACACATATGATAGGTTACACGATGTTGCTATTCA**CTTTCAAT
		Fw1	CTTTCAATCACCCTTAGTGCCTGTGGAG	
pCR8-0M	5P-3M-F8	Rw_3Mup	TAAAAGGCAACATCGTGTAACC	GTCCGAAA**GAAGGCTGAGGATGAGGTTACACGATGTTGCCTTTTA**CTTTCAAT
		Fw1	CTTTCAATCACCCTTAGTGCCTGTGGAG	
pCR8-0M	NS	Fw-WOP	CGAGCTGAAAAGCATCTTGAA	GTCCGAAA-------------------------------------CGAGCTGA
		Rw-WOP	TTTCGGACTTTTCCACCAACT	

Tab.S2: List of all primers and template plasmids used for the preparation of the different protospacer constrcuts for the self/non-self discrimination experiment. In the first two columns the names of the inverse PCR template plasmid and the name of the final construct are given. In the third column the primer name is given, followed by the primer sequence in column 4. The sequence 5´-> 3´ of the different protospacers is reported in column 5. In bold the nucleotides which correspond to the protospacer sequence, underlined the mutated 8 nucleotides of the protospacer adjacent sequence.
